# National-level effectiveness of ART to prevent early mother to child transmission of HIV in Namibia

**DOI:** 10.1371/journal.pone.0233341

**Published:** 2020-11-10

**Authors:** Andrew Agabu, Andrew L. Baughman, Christa Fischer-Walker, Michael de Klerk, Nicholus Mutenda, Francina Rusberg, Dorothea Diergaardt, Ndumbu Pentikainen, Souleymane Sawadogo, Simon Agolory, Thu-Ha Dinh

**Affiliations:** 1 US Centers for Disease Control and Prevention, Windhoek, Namibia; 2 Namibia Ministry of Health and Social Services, Windhoek, Namibia; 3 Namibia Institute of Pathology, Windhoek, Namibia; 4 US Centers for Disease Control and Prevention, Atlanta, GA, United States of America; University of North Carolina at Chapel Hill, UNITED STATES

## Abstract

**Background:**

Namibia introduced the prevention of mother to child HIV transmission (MTCT) program in 2002 and lifelong antiretroviral therapy (ART) for pregnant women (option B-plus) in 2013. We sought to quantify MTCT measured at 4–12 weeks post-delivery.

**Methods:**

During Aug 2014-Feb 2015, we recruited a nationally representative sample of 1040 pairs of mother and infant aged 4–12 weeks at routine immunizations in 60 public health clinics using two stage sampling approach. Of these, 864 HIV exposed infants had DNA-PCR HIV test results available. We defined an HIV exposed infant if born to an HIV-positive mother with documented status or diagnosed at enrollment using rapid HIV tests. Dried Blood Spots samples from HIV exposed infants were tested for HIV. Interview data and laboratory results were collected on smartphones and uploaded to a central database. We measured MTCT prevalence at 4–12 weeks post-delivery and evaluated associations between infant HIV infection and maternal and infant characteristics including maternal treatment and infant prophylaxis. All statistical analyses accounted for the survey design.

**Results:**

Based on the 864 HIV exposed infants with test results available, nationally weighted early MTCT measured at 4–12 weeks post-delivery was 1.74% (95% confidence interval (CI): 1.00%-3.01%). Overall, 62% of mothers started ART pre-conception, 33.6% during pregnancy, 1.2% post-delivery and 3.2% never received ART. Mothers who started ART before pregnancy and during pregnancy had low MTCT prevalence, 0.78% (95% CI: 0.31%-1.96%) and 0.98% (95% CI: 0.33%-2.91%), respectively. MTCT rose to 4.13% (95% CI: 0.54%-25.68%) when the mother started ART after delivery and to 11.62% (95% CI: 4.07%-28.96%) when she never received ART. The lowest MTCT of 0.76% (95% CI: 0.36% - 1.61%) was achieved when mother received ART and ARV prophylaxis within 72hrs for infant and highest 22.32% (95%CI: 2.78% -74.25%) when neither mother nor infant received ARVs. After adjusting for mother’s age, maternal ART (Prevalence Ratio (PR) = 0.10, 95% CI: 0.03–0.29) and infant ARV prophylaxis (PR = 0.32, 95% CI: 0.10–0.998) remained strong predictors of HIV transmission.

**Conclusion:**

As of 2015, Namibia achieved MTCT of 1.74%, measured at 4–12 weeks post-delivery. Women already on ART pre-conception had the lowest prevalence of MTCT emphasizing the importance of early HIV diagnosis and treatment initiation before pregnancy. Studies are needed to measure MTCT and maternal HIV seroconversion during breastfeeding.

## Introduction

The HIV epidemic remains one of the most challenging public health problems of the century. UNAIDS estimated that at the end of 2016 there were 36.7 million people living with HIV, 2.1 million were children under the age of 15 with 160,000 newly infected in 2016 [1]. Mother to child transmission of HIV (MTCT) accounts for >90% of pediatric HIV [2]. The overall HIV prevalence in general adult population 15–49 years of age is 12.3% in Namibia [1]. According to HIV sentinel survey report (2016), a nationally representative survey of pregnant women presenting at antenatal care clinics (ANC), pregnant women bear a disproportionate burden of the epidemic with a 17.2% overall prevalence and regional differences that range from 5.2% to 32.9% [3].

Namibia introduced the prevention of mother to child HIV transmission program (PMTCT) in 2002 [4]. PMTCT provides a full cascade of interventions for pregnant women including HIV counselling and testing during pregnancy, delivery, and the breastfeeding period, antiretroviral therapy (ARVs) for HIV positive women, infant HIV testing and prophylaxis, and treatment for HIV positive infants [5]. In 2013, the Ministry of Health and Social Services (MOHSS) introduced lifelong ART for all HIV positive pregnant and breastfeeding women [6]. By 2014, PMTCT services were available in 94% of all public health facilities providing ANC and maternity services [7] and the MOHSS also implemented a national strategy to reduce MTCT prevalence to less than 5% by 2017 [8].

Evaluations of the effectiveness of ART in preventing mother to child transmission of HIV have been reported in South Africa, Zimbabwe and Malawi [9–11]. However, a national-level PMTCT evaluation had not been conducted in Namibia. Routine program data and modelling (SPECTRUM) have been used to provide national level estimates, but these data have limitations for estimating program effectiveness. The Namibian PMTCT program data are aggregated monthly; there is no system in place to link the mother and her HIV exposed infant thus making it difficult to track the outcome of the PMTCT program on MTCT prevalence and maternal and child survival. SPECTRUM estimates that the MTCT prevalence at 6 weeks declined from 6.17% in 2010 to 2.00% in 2014 [12]. With the introduction of lifelong ART for PMTCT, it is critical to determine with precision beyond modeled data and program reports the effectiveness of the PMTCT program in Namibia. We sought to estimate the national level prevalence of MTCT during perinatal period (early MTCT) measured at 4–12 weeks post-delivery.

## Methods

### Study design and population

We conducted a cross–sectional clinic-based survey assessing uptake of ART among HIV infected mothers and MTCT prevalence among their HIV exposed infants 4–12 weeks of age. We recruited caregivers (hereafter referred to as mothers) and their infants from those seeking routine immunization or postnatal care services in 60 selected public health facilities between August 2014 and February 2015.

We used a two-stage sampling method to recruit participants for this study. The sampling frame for the primary sampling unit was composed of public health facilities offering routine primary health care services to mothers and children throughout Namibia. We stratified all 326 public health facilities providing routine vaccination into five strata based on quintiles of the number of first doses of DPT vaccine administered during 2012, which was used as a measure of size of the number of infants that received care at each facility. Due to the feasibility and logistics of recruiting participants from the least visited clinics in the first quintile, we excluded this stratum from our sampling frame. This stratum contained 67 clinics, which accounted for 1.4% (841/62,088) of first doses of DPT vaccine administered across all clinics.

In the first stage of sampling, within each of the four remaining sampling strata we randomly selected 15 health facilities, for a total of 60 facilities (23% of all eligible facilities). The selected facilities covered all 13 regions in Namibia. In the second stage, we recruited and enrolled all eligible and consenting caregiver-infant pairs in each facility for 19 consecutive weeks. The sampling weight for each caregiver-infant pair was calculated as the inverse of the probability of selection, defined as the probability of selecting the health facility in the first stage multiplied by the probability of selecting an HIV-exposed infant with test results in the selected health facility in the second stage. The second stage probability was based on the number of first doses of DPT vaccine administered during 2014 to reflect the total population of infants receiving care at all of the facilities at the time of sampling.

HIV-exposed infants age 4–12 weeks were identified by maternal HIV infection status. We included women with a documented HIV positive status on either the ANC card or health passport and identified new positives by testing all women with a previous negative or unknown status per national testing guidelines. We excluded severely ill infants, infants whose mothers refused consent and those younger than 4 weeks or older than 12 weeks.

### Data collection

We trained nurses at site level to identify all potentially eligible mother-infant pairs and work with trained data collectors to complete a standardized screening and interview questionnaire. We screened mother-infant pairs in two steps: (1) ensure the child was not brought to the health facility for emergency medical care and the child had not already been enrolled in the study, and (2) include only infants aged 4–12 weeks and mothers who consented for the study. For those who met these inclusion criteria, and provided written consent to participate in the study, we used the standardized questionnaire to gather sociodemographic data, clinical, treatment, and feeding data from the mother-infant pair. In addition to interviewing the mother, some clinical information was extracted from the maternal ANC cards and the infant child health passports (maternal HIV status, maternal ART use, infant HIV exposure status, and infant ARV prophylaxis).

Study nurses recorded infant HIV PCR tests and results in routine MOHSS clinic registers at each site. Confidential participant identification numbers were recorded in the study register(s) for infants/mothers enrolled in the study. The participant identification number was placed on the dried blood spot card, the laboratory request form, and the questionnaire. All interview data and laboratory results were collected on password protected smartphones and uploaded daily to a secure central database.

### HIV testing procedures

The clinic healthcare providers conducted pre- and post-test counselling and delivered HIV test results to the mother-infant pairs as per standard clinic procedures. Mothers with an unknown or previous HIV negative status were tested using parallel HIV rapid tests. Discordant results between two rapid tests were confirmed by a third rapid test (tiebreaker). All rapid HIV tests were done at the study health facilities by trained health workers.

Trained nurses collected blood samples from HIV exposed-infants whose mothers consented for infant’s HIV test on a 5-spot Guthrie card as dried blood spots. All dried blood spot specimens accompanied by routine laboratory request forms were sent to the central laboratory for HIV DNA PCR testing using COBAS AmpliPrep/COBAS TaqMan HIV-1 Qualitative Test version 2.0. Infant’s dried blood spot DNA PCR HIV test results were returned to the mother within 2–4 weeks of testing.

Prior to participation, eligible mothers were read an information sheet including study procedures and confidentiality and asked to sign written informed consent prior to participation. The participant identification numbers were used to link the mother’s and infant’s information and to provide the infant’s HIV test results to be used for clinical care. The participant identification number, name, phone number, home address and direction to home that were collected with the mother’s consent were kept in a locked box at the facility only accessed by the facility nurse and data collector. HIV test results were returned to the mother or legal guardian by clinic nurses who complied with the national guidelines. All data collectors and supervisors were trained on confidentiality procedures and signed a confidentiality agreement. All electronic survey data were encrypted. The study was approved by Namibia’s Ministry of Health and Social Services research ethical committees. The study was also reviewed in accordance with CDC human research protection procedures and was determined to be research, but CDC investigators did not interact with human subjects or have access to identifiable data or specimens for research purposes.

### Statistical analysis

We calculated descriptive statistics for maternal and infant characteristics. We calculated the weighted estimate of the national prevalence of early MTCT at 4–12 weeks and 95% confidence interval (CI). To determine importance of timing of maternal ART, we categorized self-reported maternal ART into four groups of maternal ART initiation timing: before pregnancy, during pregnancy (including delivery), after delivery, or mother never received ART. To assess additional benefit of infant prophylaxis, we also created a composite factor for maternal ART and infant ARV prophylaxis: mother received ART and infant received ARV prophylaxis, mother received ART but infant did not receive ARV prophylaxis, mother did not receive ART and infant received ARV prophylaxis, or neither received ART/ARV. For categories of each maternal and infant characteristic, we estimated the prevalence of MTCT and 95% CI.

To evaluate the association between each maternal/infant characteristic and/or PMTCT intervention and the ultimate outcome-infant HIV infection, we performed the design-based Pearson’s chi-square test. For evaluating each association, we fit a Poisson regression model to calculate prevalence ratios and 95% CIs [13]. Characteristics with a p-value less than 0.20 were considered for a multivariable Poisson regression model. Statistical significance was assessed at the 0.05 level for all analyses. All analyses were performed using Stata software version 14.2 and accounted for the two-stage survey design and sampling weights (svyset command).

## Results

### Description of study population

**Fig 1:**
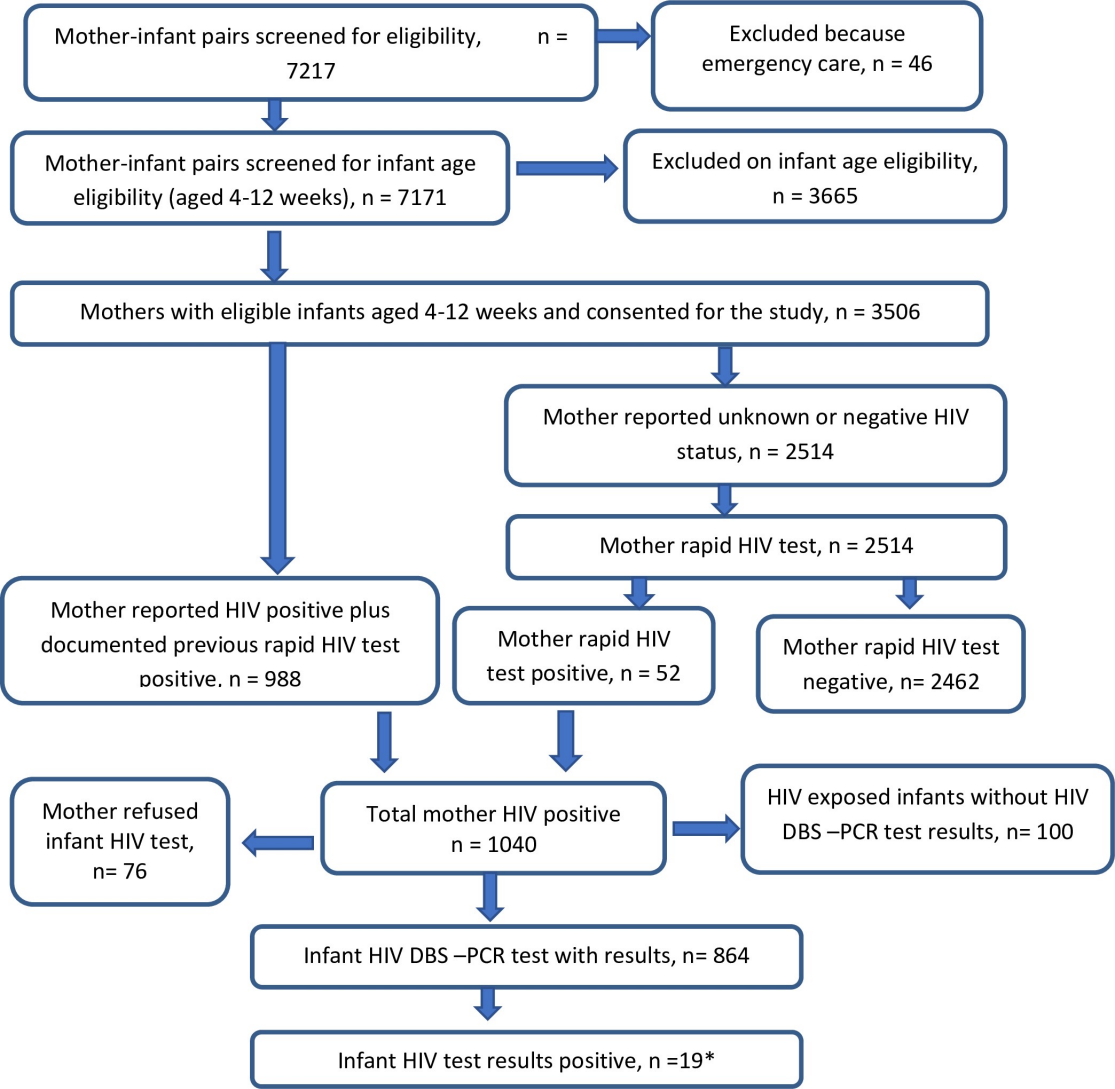
Eligibility and enrolment in Namibia PMTCT survey 2014–2015: Unweighted numbers. *4 HIV positive infants whose mothers had not provided any PMTCT information were only included in the national (overall) calculation of early MTCT prevalence and excluded in risk factor analysis.

A total of 7,217 mothers and their infants attending immunization or postnatal care services were screened in 60 study sites. Of these, 46 mother-infant pairs were excluded because their visit was for emergency care, and an additional 3,665 were excluded because the infant was not aged 4−12 weeks old, resulting in 3,506 mother-infant pairs eligible for the study (Fig 1). Among 3,506 eligible mothers, 988 had previously documented HIV positive status and 52 were newly tested HIV positive at enrollment for a total of 1,040 mothers who tested HIV positive. Among these, 864 had documented HIV test results, 76 refused consent for infant HIV testing, and 100 infants had missing HIV test results.

Of the HIV infected mothers with details of PMTCT information enrolled into the study whose infants had laboratory HIV test results (n = 860), 17.5% were aged 15–24 years and 53.2% were aged 25–34 years (Table 1). The highest proportion of the HIV infected mothers in the study reported to be single (67.4%), married/widowed/divorced (17.4%) and co-habiting (15.2%). The majority (89%) of infants were delivered vaginally. Female infants accounted for 50.2% and males were 49.8% of the HIV exposed infants enrolled in the study (Table 1).

**Table 1:** Characteristics of the study population of 860 mother-infant pairs, Namibia, 2014−2015.

Characteristic	n	Weighted % (95% CI)
Mother’s age (years)		
15–24	152	17.5 (14.7–20.8)
25–34	457	53.2 (49.8–56.7)
35–50	251	29.2 (25.3–33.5)
Marital status		
Single	576	67.4 (58.0–75.5)
Married/Widowed		
/Divorced/Separated	151	17.4 (12.1–24.5)
Cohabitating	133	15.2 (9.4–23.8)
Mother’s highest level of education		
None	103	12.4 (8.2–18.4)
Grade 1–7	307	37.0 (29.0–45.9)
Grade 8–10	356	39.2 (32.3–46.5)
Grade 11–12/	86	10.6 (6.9–16.0)
University	7	0.8 (0.4–1.8)
Unknown	1	
Weeks pregnant at first ANC visit		
1–13	234	34.7 (26.8–43.6)
14–26	398	54.1 (46.1–61.9)
27–44	87	11.2 (8.0–15.4)
Unknown	141	
Female Infants	432	50.2 (46.3–54.1)
Delivery type		
Vaginal	736	89.0 (85.2–91.8)
Caesarean	85	10.4 (7.3–14.4)
Assisted	2	0.7 (0.1–3.7)
Unknown	37	
Breastfeeding in last week		
Never	59	6.5 (4.5–9.4)
Exclusive	673	84.5 (77.9–89.4)
Mixed	61	9.0 (5.1–15.3)
Unknown	67	
Maternal alcohol use during pregnancy		
Yes	118	14.8 (10.9–19.9)
No	662	85.2 (80.1–89.1)
Unknown	80	
Mother on ART		
Yes	783	96.8 (94.5–98.1)
No	35	3.2 (1.9–5.5)
Unknown	42	
Maternal ART initiation timing		
Before pregnancy	510	62.0 (55.4–68.1)
During pregnancy	257	33.6 (27.6–40.3)
After delivery	11	1.2 (0.5–3.0)
No ART	35	3.2 (1.9–5.5)
Unknown	47	
Mother on ART–Infant receipt of NVP		
Yes–Yes	718	89.6 (81.8–94.3)
Yes–No	51	7.2 (2.8–17.1)
No–Yes	30	2.8 (1.6–4.9)
No–No	5	0.5(0.2–1.4)
Unknown	56	
Infant receipt of NVP any time after delivery		
Yes	753	92.4 (82.9–96.8)
No	56	6 (3.2–17.1)
Unknown	51	

NOTE: CI, confidence interval; ART, antiretroviral therapy; NVP, Nevirapine.

Overall maternal ART uptake was 96.8% with 62% of mothers reported starting ART before pregnancy, 33.6% during pregnancy, and 1.2% post-delivery while 3.2% never received ART. 86.5% of mothers reported never missing a dose since they started on ART. ARV prophylaxis uptake among enrolled infants was 92.4%. Both mother and infant received ARVs in 89.6% of mother-infant pairs, only mother in 7.2%, only infant in 2.8%, no ARVs for both 0.5%. Of participating mothers, 84.5% of mothers exclusively breastfed, 9.0% reported mixed feeding and 6.5% had never breastfed (Table 1).

#### Weighted national-level prevalence of early MTCT measured at 4–12 weeks of infant’s age

Based on 19 infants who tested HIV positive in the study, the weighted national early MTCT prevalence at 4–12 weeks post-partum was 1.74% (95% CI 1.00–3.01%). The design effect was 1.15, and the intraclass correlation was 0.0092. Weighting of early MTCT prevalence and univariate analysis of associated factors was done for 15 HIV infected infants with complete PMTCT information (Table 2).

**Table 2:** Univariate analysis of selected maternal and infant characteristics associated with early mother to child transmission of HIV, Namibia, 2014–2015.

	Infant			Crude	
Characteristic	n	HIV Positive	Weighted %	P-value^a^	Prevalence Ratio (95% CI)
Mother on ART					
Yes	783	11	0.9	<0.01	0.08 (0.02–0.25)
No	35	4	11.6		1.00 (Referent)
Maternal ART initiation timing					
Before pregnancy	510	6	0.8	<0.01	0.07 (0.02–0.26)
During pregnancy (including delivery)	257	4	1.0		0.08 (0.02–0.39)
After delivery	11	1	4.1		0.36 (0.04–3.40)
No ART	35	4	11.6		1.00 (Referent)
Infant receipt of NVP any time after delivery					
Yes	753	12	1.0	0.01	0.26 (0.09–0.73)
No	56	3	4.0		1.00 (Referent)
Mother on ART–Infant receipt of NVP					
Yes–Yes	718	9	0.8	<0.01	0.03 (0.01–0.22)
Yes–No	51	2	2.7		0.12 (0.02–0.79)
No–Yes	30	3	9.6		0.43 (0.03–5.32)
No–No	5	1	22.3		1.00 (Referent)
Mother’s age (years)					
15–24	152	3	0.9	0.01	1.00 (Referent) ^b^
25–34	457	12	1.9		0.53 (0.32–0.87)
35–50	251	0	0.0		0.23 (0.07–0.72)
Parity					
1	95	5	2.6	0.08	1.00 (Referent)
2	221	2	0.4		0.16 (0.03–0.89)
3	212	5	2.0		0.80 (0.23–2.80)
4+	295	3	0.7		0.27 (0.06–1.31)
Breastfeeding in last week					
Never	59	2	2.5	0.40	1.00 (Referent)
Exclusive	673	10	1.1		0.45 (0.09–2.19)
Mixed	61	3	2.0		0.81 (0.17–3.79)
Mother’s highest level of education					
None	103	1	0.6	0.82	1.00 (Referent)
Grade 1–7	307	4	1.1		1.92 (0.20–18.8)
Grade 8–10	356	8	1.5		2.66 (0.27–26.2)
Grade 11–12 /Univ. ^c^	93	2	0.9		1.50 (0.09–26.5)
Delivery type					
Vaginal	736	14	1.3	0.63	1.00 (Referent)
Caesarean/Assisted^d^	87	1	0.8		0.59 (0.07–5.24)
Maternal report of any alcohol use during pregnancy					
Yes	118	4	2.6	0.11	2.38 (0.78–7.28)
No	662	11	1.1		1.00 (Referent)
Disclosure of maternal HIV status to a family member or a friend					
Yes	687	11	1.2	0.67	1.00 (Referent)
No	136	4	1.5		1.32 (0.36–4.80)
Knowledge of mode of MTCT					
Breastfeeding	137	3	2.1	0.43	2.06 (0.34–12.6)
During pregnancy	97	2	1.0		1.00 (Referent)
During childbirth	422	8	1.2		1.16 (0.20–6.61)
Other	82	1	0.5		0.47 (0.03–7.14)
Don’t know	19	1	4.5		4.44 (0.28–70.1)

NOTE: ART, antiretroviral therapy; NVP, Nevirapine.

^a^Pearson’s design-based chi-square test of the null hypothesis of no association between the characteristic and infant HIV infection.

^b^Mother’s age was treated as a continuous variable and was statistically significant (t-test, p = 0.02). The midpoints of the age groups were used to derive prevalence ratios.

^c^The seven infants in the university subgroup were combined with the 86 infants in the grade 11–12 subgroup because none of the infants in the university subgroup tested positive for HIV.

^d^The two infants in the assisted delivery subgroup were combined with the 85 infants in the Caesarean subgroup because neither of the infants in the assisted delivery subgroup tested positive for HIV.

**Fig 2:**
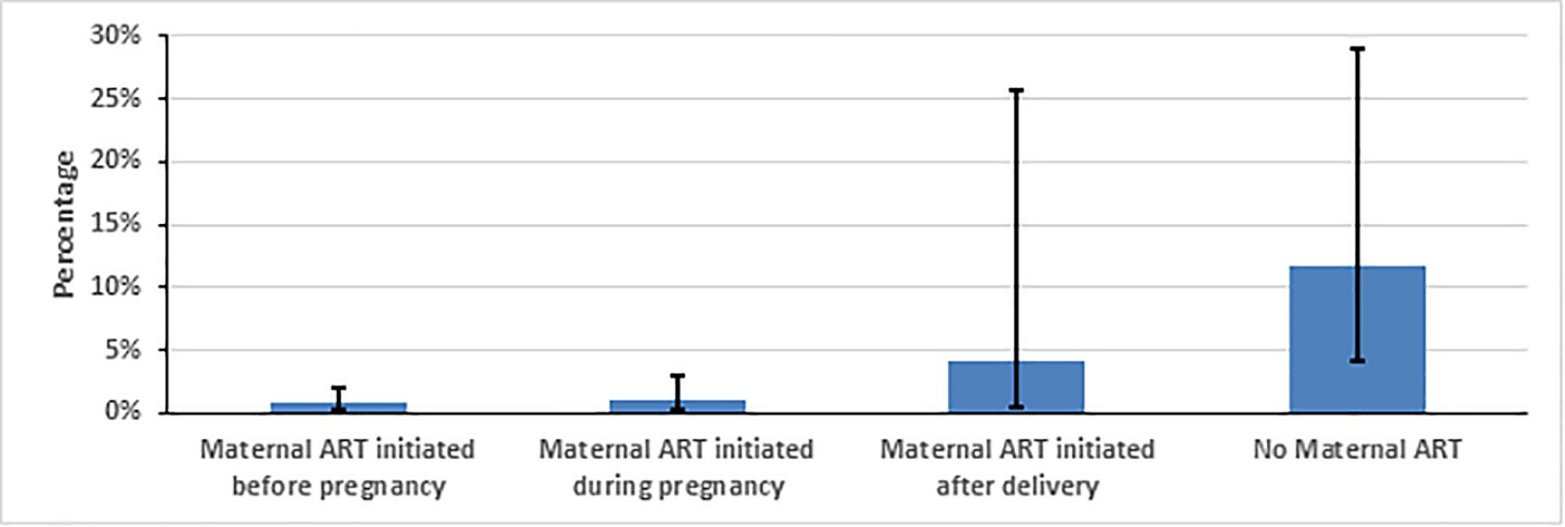
Weighted early mother to child transmission of HIV measured at 4–12 weeks post-partum by maternal ART initiation time, Namibia, 2014–2015.

Mothers on ART were 92% less likely to infect their infants compared to those not on ART (prevalence ratio: 0.08, 95% CI: 0.02−0.25) (Table 2). Timing of mother’s ART initiation also had a strong association with HIV transmission: infants born to mothers who started ART before pregnancy and during pregnancy had lower prevalence of HIV infection from their mothers, 0.78% (95% CI, 0.31% -1.96%) and 0.98% (0.33%-2.91%), respectively. The prevalence of infant HIV infection increased to 4.13% (0.54%-25.68%) when mother was initiated ART after delivery and 11.62% (4.07%-28.96%) when mother never received ART (Table 2 and Fig 2).

**Fig 3:**
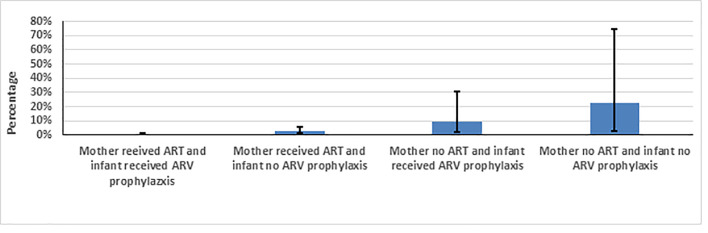
Weighted early MTCT prevalence measured at 4–12 weeks post-partum by maternal ART and infant ARV prophylaxis, Namibia, 2014–2015.

The lowest MTCT prevalence of 0.76%, (0.36%-1.61%) was achieved when mother received ART and infant received ARV prophylaxis (Table 2 and Fig 3). MTCT prevalences were 2.67% (1.26%-5.54%) when only mother received ART, 9.62% (2.47%-30.91%) if only infant received ARV prophylaxis, and 22.32% (2.78%-74.25%) when neither mother nor infant received ARVs.

When mother’s age was treated as a continuous variable, we found a downward trend of early MTCT prevalence with increasing mother’s age. Mothers’ aged 25–34 years had higher MTCT prevalence (1.98%) than older mothers aged 35−50 years (Table 2). Parity was also negatively correlated with the early MTCT prevalence. The weighted early MTCT prevalence was 3.8% if it was the mother’s first pregnancy and 3.2% if 1^st^ delivery.

#### Multivariable analysis of maternal and infant characteristics associated with mother to child transmission of HIV

Maternal and infant treatment characteristics, mother’s age, and parity were considered for multivariable analysis (Table 2). Because only 15 infants tested positive for HIV, for parsimony we included in a multivariable the two binary treatment characteristics, mother’s ART use and infant’s receipt of NVP, and mother’s age as a continuous variable. Parity was not included because it was strongly associated with mother’s age. After adjusting for mother’s age, mother’s ART use and infant’s receipt of NVP remained strong, independent predictors of early MTCT (Table 3). Mothers who were on ART were 90% less likely to have an infant who tested positive for HIV (prevalence ratio: 0.10, 95% CI: 0.03−0.29), and infants who received NVP at any time after delivery were 68% less likely to test positive for HIV (prevalence ratio: 0.32, 95% CI: 0.10−0.998). In the multivariable model, no interaction was detected between mother’s ART use and infant’s receipt of NVP.

**Table 3:** Multivariable analysis of mother’s receipt of ART, infant’s receipt of NVP, and mother’s age with early mother to child transmission of HIV.

	Adjusted Results^a^	
Characteristic	P-value^b^	Prevalence Ratio (95% CI)
Mother on ART		
Yes	<0.01	0.10 (0.03–0.29)
No		1.00 (Referent)Infant receipt of NVP any time after delivery
Yes	0.05	0.32 (0.10–0.998)
No		1.00 (Referent)
Mother’s age (years)		
15–24	0.05	1.00 (Referent)^c^
25–34		0.62 (0.39–0.98)
35–50		0.33 (0.12–0.96)

NOTE: ART, antiretroviral therapy; NVP, Nevirapine.

^a^Fifty-six (6.5%) mother-infant pairs were excluded from the model because of missing information on whether the mother was currently on ART or whether her infant received NVP. The model includes the characteristics listed in the table.

^b^Adjusted Wald test of the null hypothesis of no association between the characteristic and infant HIV infection.

^c^Mother’s age was treated as a continuous variable in the model. The midpoints of the age groups were used to derive prevalence ratios.

## Discussion

Our study finding of 1.74% MTCT prevalence at 4–12 weeks is consistent with the MTCT modelled prevalence in SPECTRUM of 2% at 6 weeks post-delivery in Namibia in 2014 [12]. However, it is half of the early MTCT prevalence (3.5%) measured at 4–8 weeks in South Africa in 2010 [9] and 3.7% at 4–26 weeks in Malawi [11]. The lower MTCT prevalence of 1.74% in Namibia could be due to high coverage (96.8%) of maternal ART among the study participants compared to only 29% on ART and 71% on maternal ARV prophylaxis in the South African study [9]. This finding underlines the importance of high ART uptake for all HIV positive pregnant women.

As of 2015, Namibia reduced early MTCT to 1.74% from 6.17% in 2010. Without any PMTCT interventions, the overall prevalence of infection was estimated to be 25–40% [14]. The 22.3% early transmission prevalence we observed in our study among the few mother-infant pairs who did not access antiretroviral treatment is an indication that overall prevalence at the end of breastfeeding period would be high. Comparing the early MTCT modelled prevalence in SPECTRUM of 6.17% in 2010 [12] with our observed eMTCT prevalence of 1.74%, assuming 68,218 births in 2014 [15] and 16.9% HIV prevalence in 2014 [16], we estimate there were 11,529 HIV pregnant women, and therefore, the estimated number of incident infant HIV infections declined from 712 in 2010 to 201 in 2014. This 72% reduction in mother to infant transmission over 4 years was achieved with 96.8% maternal ART and 92.4% infant ARV prophylaxis coverage in a predominantly breastfeeding population.

Like other studies that showed significant difference in MTCT prevalences with duration of exposure to ART, we found that transmission prevalence was lowest (0.78%) when pregnant women initiated ART before pregnancy while it was 1.4% in Malawi [11] and 1.2% at 6 weeks post-delivery in Zimbabwe [10]. The lower MTCT prevalence when ART started pre-conception is likely a result of majority of mothers having viral suppression before delivery. We also found that MTCT was slightly higher (0.98%) when mother started ART during pregnancy, however, our study result was lower than 2.4% in Zimbabwe [10] and 3.4% in Malawi [11]. These differences are likely due to timing of initiation of ART during pregnancy. In our study 34.7% of women started ANC during 1^st^ trimester and 54.1% in 2^nd^ trimester compared to 20% and 55.7% respectively in Zimbabwe study [10]. Considering that studies have shown that HIV positive mothers who received <4 weeks of ART before delivery have higher MTCT [17], the MTCT prevalences among those initiating ART during pregnancy would have been lower had they all started ART before pregnancy or within 1^st^ trimester of pregnancy.

The high MTCT prevalence of 4.13% when mother was initiated ART post-delivery in our study was similar to 1.0–5.0% reported in Malawi [18, 19]. However, post-partum initiation of ART was 3 times more protective than no ART at all which had a MTCT prevalence of 11.62%.

We also assessed whether other maternal and infant factors were associated with prevalence of HIV transmission from HIV infected mothers to their infants. Parity negatively correlated with the early MTCT prevalence, although these trends were not statistically significant. The weighted early MTCT prevalence was 3.8% if it was the mother’s first pregnancy and 3.2% if 1^st^ delivery. None of the other non-ART/ARV factors were associated with HIV infection in the infant. However, we know that these factors have been shown to have an effect on MTCT as they are associated with PMTCT service quality: service uptake, time of ART initiation, adherence to ART, retention in ART and maternal disease progression [9, 20–23]. The finding of no association was likely influenced by the low number of HIV infected infants in the study.

**Implications of our findings to eMTCT**

Importance of early case identification among childbearing women and linkage to ART in line with treat all approach before conception maximizes impact of PMTCT.

Retesting of previously negative pregnant and breastfeeding women is critical to determine seroconversion rates and to offer opportunity to initiate ART if HIV infected.

Cohort monitoring is needed to measure the impact of PMTCT program on MTCT by the end of breastfeeding period.

## Limitations

There were several limitations in our study. Excluding small volume clinics may have affected the national representativeness of our study; however, small volume clinics excluded from our sampling frame represented only 1.4% of patients at all clinics. In addition, some eligible mother-infant pairs may have been excluded in our study because infants were severely ill, or they attended private clinics for their health services. Our study being facility-based it excluded eligible mother-infant pairs who did not attend any health facility for immunization or other health services. The small number of HIV positive infants led to wide confidence intervals and limited our assessment of risk factors for early MTCT, including timing of maternal ART and additional benefit of infant ARV prophylaxis. Accurate information on the exact PMTCT regimens, duration of these regimens, and adherence could not all be adequately verified in the absence of complete documentation, as our data was largely self-reported and subject to recall bias. Data on other factors should also be viewed with caution as they were likely subjected to the same recall bias. In our study, 16.9% of HIV exposed infants had no HIV test results, which may have led to a selection bias; however, an analysis of characteristics of mothers with infants who had no HIV test results, such as age and parity, showed that they were not different from those with test results.

## Conclusions

As of 2015, Namibia achieved MTCT prevalence of 1.74%, measured at 4–12 weeks post-delivery. Women already on ART pre-conception and those initiated during pregnancy had lower prevalence of MTCT emphasizing the importance of early HIV diagnosis and treatment initiation before and earlier during pregnancy to achieve the elimination of MTCT in a breastfeeding population with high HIV prevalence. There is need to ensure mother-infants pairs continue receiving high quality PMTCT services and sustain high levels of maternal ART adherence and viral load suppression throughout the breastfeeding period. Studies are also needed to measure MTCT and maternal HIV seroconversion during breastfeeding.
